# Supplementary material for: High Input of Nitrogen Fertilization and Short Irrigation Frequencies Forcefully Promote the Development of Verticillium Wilt of Olive
Source: Plants (Basel). 2022 Dec 16;11(24):3551. doi: 10.3390/plants11243551 (PMC9781960; doi:10.3390/plants11243551)
Supplement: Supplementary file 1 [file plants-11-03551-s001.zip › plants-2043791-supplementary.pdf]

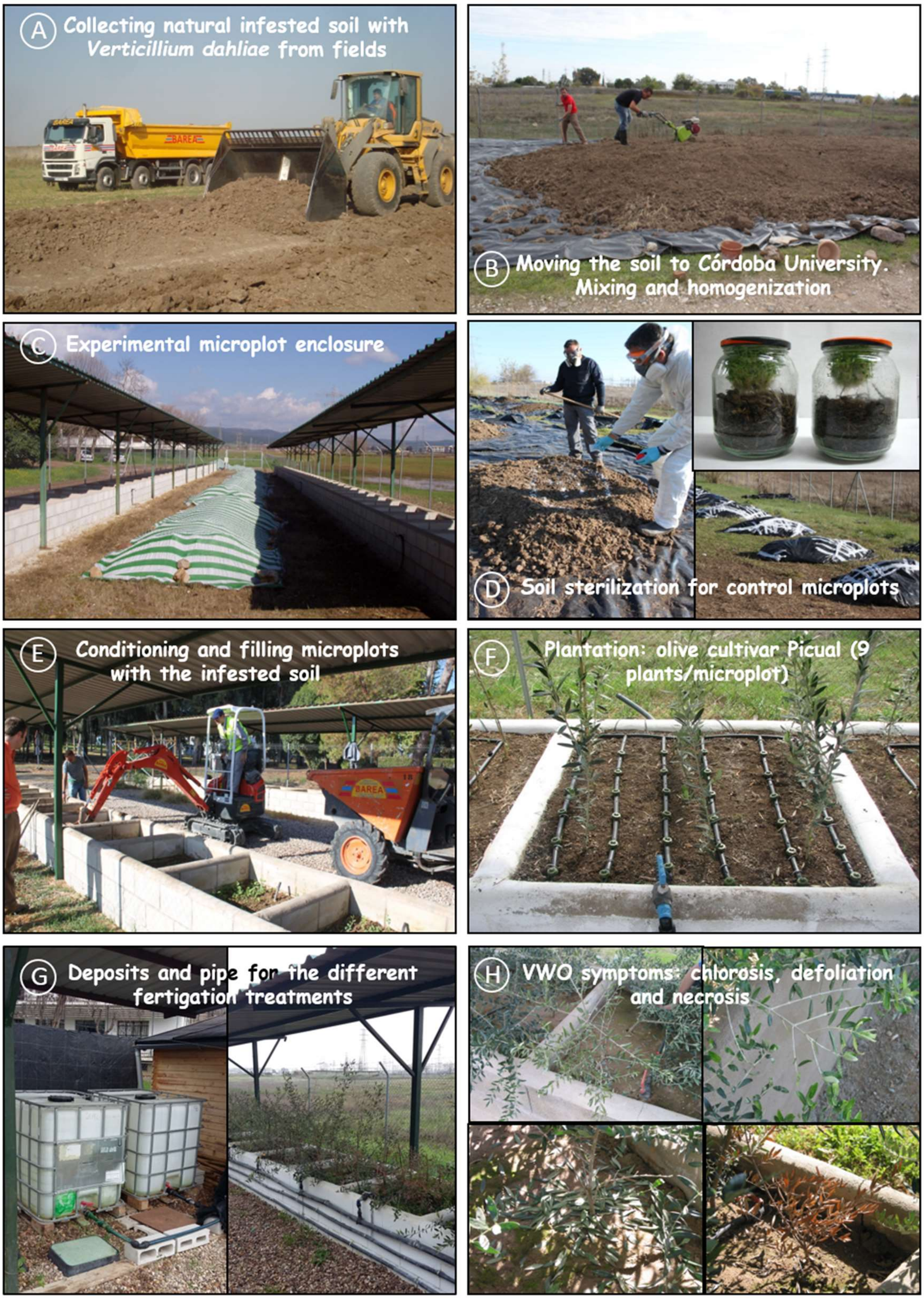

Figure S1. Overview of the establishment of microplots with soils naturally infested with *Verticillium dahliae,* and the symptoms of the disease, in a trial where the influence of fertilization and its interaction with irrigation frequency on the development of Verticillium wilt of olive (VWO) were evaluated in the olive cultivar Picual.
